# Antiseptics

**Published:** 1883-02

**Authors:** 


### SELECTIONS.

### Antiseptics.

In the *New York Medical Journal*, Dr. Willard Parker, speaking of carbolic acid in the treatment of wounds, says he singles it out for the reason that it is better known by the profession, and more closely bound up, so to speak, with what we know as Listerism, than the other drugs that are used for like purposes. It is his decided conviction that the use of carbolic acid is a valuable adjuvant in surgical practice. As a mere disinfectant, it is perhaps in no way superior to a number of other agents; probably inferior to some. But as an antiseptic and detergent, it is not surpassed by any other substance. Its efficiency in averting inflammatory complications is mainly due to its germicidal properties. It is valuable also as a topical stimulant to ill-conditioned suppurating surfaces, such as the walls of cold abscesses and the like. It is especially needed when wounds have to be dressed in a vitiated atmosphere.

Dr. Robert F. Weir, after an individual experience of nearly six years in the wards of the Roosevelt, New York, and Bellevue Hospitals, depends in the main on carbolic acid as a germicide. Yet it has the disadvantage of occasionally acting toxically on patients. Next to carbolic acid, he regards iodoform and bichloride of mercury as the most secure germicides. He uses iodoform by insufflation upon the wounds, also iodoformized gauze, which is made by impregnating the gauze, previously washed with 50 parts iodoform, 250 parts ether, and 750 parts alcohol. This gives a ten per cent. gauze. In the employment of bichloride of mercury as an antiseptic, about six grains to the pint are used, or a solution of similar strength is irrigated over the wound. Sponges and compresses are also used in this solution. Gauze also is dipped in a solution of forty grains of corrosive sublimate to a pint of alcohol, to which is added one and a half ounces of glycerine. Under such dressings, within a short time, an amputation at the hip joint has passed satisfactorily towards complete recovery. The additional advantages of the sublimate dressing are its freedom from odor, its slight effect upon the hands, its cheapness and easy preparation. With these three antiseptics all wounds under his care are now being treated, preference being given to carbolic acid. Volkmann reiterates his remark, that no suppuration will take place if septic infection is prevented. Esmarch and Nussbaum pronounce a surgeon criminal in neglecting antiseptic measures.

Dr. William Bull, surgeon to St. Luke's and to Chambers Street Hospitals, London, does not think the antiseptic treatment generally enough adopted by practitioners as desirable. It is admitted to be indispensable for all important operations and wounds, and in wards where many patients are congregated, but is thought unnecessary or of less value in the minor wounds which occur in everyday practice. Now, it is a fact that these minor wounds may be the starting-point of the most serious inflammations, and it is hence most important so to treat them as to prevent such inflammations. It is to wounds of the scalp and hands, and small wounds in the vicinity of larger joints or overlying the pleural or peritoneal cavity, that he directs its use.

